# Resilience and Well-Being Among Children of Migrant Parents in South-East Asia

**DOI:** 10.1111/j.1467-8624.2012.01810.x

**Published:** 2012-09-11

**Authors:** Lucy P Jordan, Elspeth Graham

**Affiliations:** University of Southampton; University of St. Andrews

## Abstract

There has been little systematic empirical research on the well-being of children in transnational households in South-East Asia—a major sending region for contract migrants. This study uses survey data collected in 2008 from children aged 9, 10, and 11 and their caregivers in Indonesia, the Philippines, and Vietnam (*N* = 1,498). Results indicate that while children of migrant parents, especially migrant mothers, are less likely to be happy compared to children in nonmigrant households, greater resilience in child well-being is associated to longer durations of maternal absence. There is no evidence for a direct parental migration effect on school enjoyment and performance. The analyses highlight the sensitivity of results to the dimension of child well-being measured and who makes the assessment.

South-East Asia is a major exporter of labor migrants, many of whom leave children behind in the country of origin while they work overseas on short-term employment contracts, thus creating a global network of transnational families. These migrations are often motivated by a desire to secure a higher standard of living for children and other kin, although it is increasingly recognized that there may be social as well as financial costs to this strategy. To date there has been little systematic empirical research on the well-being of children in transnational households.

The experience of immigrant children residing in host countries such as Canada and the United States is better researched in comparison to the experiences of children left behind in sending communities, with studies suggesting that first-generation immigrant children exhibit fewer emotional and behavioral problems compared to nonimmigrant children despite significant economic disadvantage, although these indicators of resilience decline over time (Beiser, Hou, Hyman, & Tousignant, 2002; Georgiades, Boyle, & Duku, 2007; Harker, 2001; Stevens & Vollebergh, 2008). The comparatively better outcomes for immigrant youth are often attributed to a few main causes, including the healthy migrant effect that selectively brings the “brightest and the best” immigrants into host countries (Razum, Zeeb, & Rohrmann, 2000; Rubalcava, Teruel, Thomas, & Goldman, 2008) and family process variables that may serve as protective factors (Harker, 2001; Suarez-Orozco & Qin, 2006). While research on immigrant children can inform theoretical explanations for the well-being of children left behind, the psychological development of left-behind children is likely to differ from the experiences of immigrant children. Left-behind children continue to experience familiarity in their quotidian life while simultaneously experiencing the rupture of parental departure and absence. How these experiences differ is an under-researched area of inquiry.

This study builds on recently completed work on left-behind children in South-East Asia, which used a standardized screening tool to investigate their psychological well-being (Graham & Jordan, 2011). The current investigation focuses on more subjective measures and incorporates two perspectives on child well-being: (a) that of 9-, 10- and 11-year-old children themselves, and (b) that of the children’s main caregivers. The aims of the article are twofold. The first is to investigate whether or not parental migration overseas compromises the well-being of children left behind, and the second is to examine both the child perspective and the adult caregiver perspective. This comparison is important because existing studies often rely on one or the other (Episcopal Commission for the Pastoral Care of Migrants and Itinerant People -CBCP/Apostleship of the Sea -Manila, Scalabrini Migration Centre, & Overseas Workers Welfare ECMI–CBCP/AOS–Manila, SMC, & OWWA, [2004]; Graham & Jordan, 2011) and discrepancies between the two would raise questions about how best to measure child well-being. Also, caregiver reports may reflect culturally informed perceptions, or ideologies, more strongly than those of the children and the well-being of the caregivers themselves may also be influenced by their personal experiences of the migration event. Furthermore, the age range of the children in this study reflects a relatively neglected population—that of children in middle childhood, a pivotal turning point as “children begin to navigate their own ways through societal structures, forming ideas about their individual talents and aspirations for the future … [with] major implications for their success as adults” (García Coll & Szalacha, 2004: 81). Understanding developmental processes for this age group is critical to supporting the passage through adolescence into adulthood for emergent family forms such as the transnational family.

## Migration, Resilience, and Well-Being of Children

While there is growing interest in the circumstances of children of migrant parents who remain in their countries of origin, theoretical understanding remains underdeveloped. Household migration is typically considered a livelihood strategy that is taken up due to economic deficiencies (Laudy & Stark, 1988; Stark & Lucas, 1988). Debate remains about whether labor migration is an effective long-term poverty alleviation strategy at the household and national levels (Adams & Page, 2005) even though research has indicated, in general, rising standards of living for households that receive remittances from family migrant laborers (Gulati, 1993; Hadi, 1999; Sofranko & Idris, 1999). There is a general tendency, however, to articulate concerns about children of migrant parents who may suffer emotional costs even if their material well-being is improved, and this concern is expressed with greater emphasis when the migrant parent is the mother, as is increasingly common for many sending countries throughout the world (Parreñas, 2001).

Within South-East Asia, there is limited research on the well-being of children of overseas migrant parents, with studies on the Philippines being one of the few exceptions. For example, a survey of Filipino school-age children (*N* = 1,443) found that migrant’s children tended to have better physical health than nonmigrant’s children (ECMI–CBCP/AOS–Manila et al., 2004). Differences based on the gender of the migrant parent were also noted. In particular, nonmigrant’s children fell sick more frequently than children of father migrants, although a slightly higher proportion of children of mother migrants was susceptible to common ailments and loss of appetite. Children of mother migrants also appeared to have poorer psychological well-being, with more reporting themselves as being unhappy, anxious, and lonely, although this may be mitigated by communication with the migrant parent because a greater frequency of communication was found to be associated with better well-being outcomes for left-behind children. This study provides some insight into patterns of well-being for children of migrant parents, but the reported analyses were purely descriptive, disallowing any multivariate explanations.

Other studies in the Philippines have made use of retrospective data from young adult children of migrant parents to elaborate on the challenges these children faced (Parreñas, 2001, 2005, 2008). Parreñas (2001) found that adult children identified emotional insecurities (in reference to *mothers*) and emotional gaps (in reference to *fathers*) resulting from parental migration (Parreñas, 2008). The notion of an emotional gap between migrant parent and left-behind child is echoed in other research on internal migrants in Vietnam (Locke, Nguyen, & Nguyen, 2012) and sending areas in Latin America (Hondagneu-Sotelo & Avial, 1997). Parreñas rightly points out that for children left behind in the Philippines, “staying together” and keeping the family “whole” are worth much more than achieving financial security. Children, however, can make such sweeping claims more easily, because the material security provided by migrant parents affords them “the luxury of demanding greater emotional security; *it is highly unlikely that impoverished children would make similar demands”* (emphasis added) (Parreñas, 2001, p. 376). In lower income countries, the material gains as a result of international remittances may not replace the emotional loss of parental separation, but chronic poverty may be worse in the long run for child development. This highlights the need for comparative studies, along with the importance of examining how the gender of the migrant parent may influence dimensions of child well-being in transnational households.

Recent studies in China offer some insight into the situation of left-behind children, although few of these findings have been published outside of China, making the research largely inaccessible to non-Mandarin speakers. The dynamics of migration in China differ from those of transnational households in South-East Asia as migration is largely internal, that is, within the national boundaries, although the household registration system, or Hukou, makes it difficult, but not impossible, for families to migrate with their children into urban areas. One recent study using the Strengths and Difficulties Questionnaire (SDQ), an international brief screening instrument for psychological well-being widely used in many countries including Asia (Du, Kou, & Coghill, 2008; Goodman, Renfrew, & Mullick, 2000; Mullick & Goodman, 2001), is particularly relevant to our research. Fan, Su, Gill, and Birmaher (2010) found that left-behind children in one Chinese province were at greater risk of developing emotional problems, especially if the parent left when the child was young or was gone for longer durations. Additionally, caregiver characteristics influenced child well-being, with children in the care of younger caregivers and those with lower levels of education at greatest risk.

During recent decades scholarship regarding the “feminization” of migration has increased significantly (e.g., Asis, Huang, & Yeoh, 2004; Piper, 2008), and with good reason as female participation in migration, epitomized by the Filipina maid in the Middle East or nurse in the United States, represents a relatively recent trend in global labor migration. Countries within South-East Asia illustrate the feminization of international labor migration to varying degrees. The Philippines and Indonesia are often highlighted as examples of growing feminization with some estimates suggesting that females constitute over 70% of the deployment of overseas foreign workers in both countries (Asis, 2003). However, within other countries in the region such as Vietnam and Thailand, the percentage of international female migrants is much lower at around 20% (Jatrana, Toyota, & Yeoh, 2005). While there is no reliable evidence on the number of children left in the sending communities by these migrants, the increased feminization is linked, unsurprisingly, to concern about the well-being of left-behind children as their mothers take up care work in the wealthier nations of the world (Hochschild, 2003). This is important because it raises questions about whether child well-being outcomes do differ according to the gender of the migrant parent. Any such differentiation could arise from a variety of factors, including differences in monetary remitting patterns and caregiving arrangements for children in the sending country. It could also be associated with traditional gender ideologies where, in countries such as the Philippines, the “ideological constructs of feminine identity still follow the cult of domesticity” (Parreñas, 2001, p. 381), a reality that can “aggravate the emotional strains of mothers and children in transnational [mother migrant] families” (Parreñas, 2001, p. 362). The powerful influence of social prescriptions about the gendered nature of caregiving may be cause for concern regarding child well-being if children internalize negative self-conceptualizations because of living in the “wrong kind” of family (Parreñas, 2010). While Parreñas has begun to outline a theoretical framework, there is a notable lack of empirical testing of the concepts within this field of study. The literature on China, for example, tends to be silent on the topic of the effect of the migrant parent’s gender on child well-being, which may be due, in part, to the predominance of children left behind with elderly caregivers (Jia & Tian, 2010).

Previous research indicates that transnational family arrangements exact an emotional cost for children who remain in the sending country even while their material security is enhanced. However, much of the present knowledge is based on descriptive analysis of bivariate relations, and on relatively small scale retrospective studies. There are a number of studies on Latin American sending countries, but their findings may not be directly applicable to South-East Asia due to differences in culture and migration patterns (Dreby, 2007; Orellana, Thorne, Chee, & Lam, 2001). In Latin America, chain migration, whereby children follow parents, is much more common than in South-East Asia where the settlement of children in host countries is generally prohibited. Comparative study within Latin America (Sana & Massey, 2005) highlights the need for caution in drawing conclusions without considering cultural diversity within geographically proximate regions. The Philippines, as a major exporter of overseas foreign workers, is the most frequently studied country in South-East Asia, although there are a few studies from other countries in the region such as Indonesia (Hugo, 2002) and Thailand (Jampaklay, 2006). The lack of comparative empirical work inhibits understanding of more universal effects of parental migration on children, including whether the trade-off for greater material security may be the emotional costs of growing up without one or both parents.

## Research Framework and Questions

The significant gap in theoretical understanding of the experiences of children of migrant parents who remain in sending countries leads us to draw initially on the extant literature about the experience of immigrant children in host societies, in particular adapting the model developed by García Coll et al. (1996; García Coll et al., 2004) to aid our analysis. Position in the social status hierarchy is an important influence on children in middle childhood who are participating increasingly in wider social settings and, relative to their peers in a host society, immigrant children are in a minority position because of their “foreignness.” Over time, they may become susceptible to the influence of negative social status positionality that is common for racial and ethnic minorities in most host countries. Family process measures such as cohesion within the immigrant family are considered protective and are associated with well-being and resilience in immigrant children, and erosion of this cohesion over second and third generations is one of the significant contributing factors to decreased well-being over time among immigrant youth (Harker, 2001). We propose that the influence of social status may operate differently for children who remain in sending countries when one or both of their parents migrate. Their everyday lives within wider ecosystemic levels (e.g., school, neighborhood) remain much the same. The biggest changes occur within the family at the household level, in direct contrast to immigrant children who travel with (or join) their family in a foreign host country where the biggest changes occur beyond the household level. The social status of left-behind children may be positively enhanced relative to their peers through increased wealth–enabling access to popular consumer goods, even as it may be negatively impacted when children take on a new social status as a “child of a migrant.” This new status may be particularly damaging if the migrant is the mother when predominant cultural expectations about gender and caregiving prescribe maternal proximity as essential for emotional nurturance. Within many Asian cultures, the importance of multigenerational kin networks is well documented (Frankenberg, Lillard, & Willis, 2002) and the normative practice of extended family living arrangements may help to mitigate some of the transitions within transnational households. Household structure and family process may provide a buffer from some of the negative impacts of parental absence on left-behind children, but we do not expect to find uniform differences in well-being between children of migrant parents and those who are living with both parents across the study countries. In a previous study (Graham & Jordan, 2011) using standardized measures from the SDQ we found that children of *migrant fathers* in Indonesia and Thailand were more likely to have poor psychological well-being compared to children in nonmigrant households. This finding was not replicated for the Philippines or Vietnam. We also found evidence that children of migrant parents in the Philippines had a *relative advantage* in terms of psychological well-being compared to their peers in nonmigrant households. The current study employs other, more subjective, measures of child well-being based on child self-report and adult caregiver report. This strategy extends the range of well-being dimensions examined to provide some insight into the sensitivity of results to the particular measure used. The study seeks to answer the following related research questions:

Question 1: Is maternal and/or paternal migration associated with decreased child well-being?
Question 2: Does the relative social status of children moderate the influence of parental migration status on child well-being?
Question 3: To what extent do caregiver and child assessments of child well-being differ?

## Method

The analysis used data from a cross-sectional baseline study of Child Health and Migrant Parents in South-East Asia (CHAMPSEA) which collected survey data for approximately 4,000 index children and their households across four study countries in the region (Indonesia, the Philippines, Thailand, and Vietnam). Index children were selected from one of two age groups: one in early childhood aged 3, 4, and 5 years and the other in middle childhood aged 9, 10, and 11 years. Survey instruments included a household questionnaire administered to the responsible adult in the household, a carer questionnaire administered to the main caregiver of the index child (for some households, this is the same person as the responsible adult), and an older child questionnaire administered to the children aged 9, 10, and 11 years. Data for the present study were drawn mainly from the carer and the older child questionnaires, with the exception of household structure information which was drawn from the household questionnaire.

In the absence of suitable sampling frames for migrant parent households, CHAMPSEA employed a three-stage flexible quota sampling strategy adapted from “sentinel site surveillance” methods (Byass et al., 2002; Wilson, Huttly, & Fenn, 2006). The resultant sample is not nationally representative but detailed protocols were designed to allow future replication. Only households including a child in one of the two age groups of interest and fulfilling specified criteria in relation to parental migrant status were eligible for the study. Single-parent households were excluded, as were households where one or both parents were internal migrants. Children in both nonmigrant and transnational households were sampled where the household migration status had not changed for a continuous period of at least 6 months before interview. The 6-month period was chosen to provide findings comparable with a previous study in Sri Lanka (Save the Children, 2006) and because it was considered a sufficient length of time to capture nontransient effects of parental migration. Transnational households were purposively oversampled to fulfil the study’s objectives of comparing the health and well-being of children in different types of transnational households (father migrant, mother migrant, and both parents migrant) against the benchmark of children living with both parents in the same communities. Quotas ensured that at least half of the index children lived in transnational households and approximately equal numbers of young and older, and male and female children were selected. Only one index child was identified in each eligible household (see Graham & Jordan, 2011, for a detailed discussion of the sampling strategy).

All interviewees in households recruited to the survey gave informed consent (assent in the case of the children) and interviews were conducted in local languages. Questionnaires were first compiled in English and then subject to translation-back translation to ensure consistency of meaning in local versions. Experienced interviewers recruited in each study country were given standard training across the study country sites and ethics approval was obtained from the University of St. Andrews, National University of Singapore, Scalabrini Migration Center (Philippines), Center for Population and Policy Studies, Gadjah Mada University (Indonesia), Institute for Population and Social Research, Mahidol University (Thailand), and Asia-Pacific Economic Center (Vietnam). The sample from Thailand was excluded from the present study because a major focus is on differences in child well-being across different types of transnational household and, despite a concerted effort, only three mother migrant households meeting the CHAMPSEA selection criteria were identified. The following analyses therefore used data from three of the four study countries (Indonesia, the Philippines, and Vietnam).

### Sample for Analysis

The selected sample comprises children aged 9, 10, and 11 and their households in Indonesia, the Philippines, and Vietnam (*n* = 1,523). The final analytical sample consisted of 1,498 children and their households after listwise case deletion for missing data. This is a reduction of less than 2% and well within an acceptable range for missing data; thus, no data have been imputed. Table 1 displays the descriptive characteristics of the analytical sample.

**Table 1:** Proportions and Means (Standard Deviations) of Dependent, Independent, and Control Variables by Household Migration Status (N = 1,498)

			International migrant			
				
Characteristics	Full sample	Nonmigrant parents	Father	Mother	Both parents	Test statistic
Child level						
General happiness (SR)	80.91	85.43_a_	78.18	75.2_a_	78.87	19.34***
Enjoyment at school (SR)	85.71	86.13	85.15	85.64	84.51	
Child is happy (CR)	57.34	60.64_a_	57.88	49.35_a_	64.79	14.84**
School performance (SR)	29.97	28.29	32.12	31.33	29.58	
School class position (CR)	42.26	40.76	42.73	43.6	47.89	
Child age						
9	36.52	35.57	35.15	37.86	45.07	
10	35.31	37.25	34.24	33.16	32.39	
11	28.17	27.17	30.61	28.98	22.54	
% children female	51.13	50.42	50.3	52.22	56.34	
Knowledge of other transnational households	78.64	75.35_ab_	83.64_a_	78.59	88.73_b_	13.81**
Household level						
Duration of maternal migration	0.12 (.08)	0.02 (.09)_ab_	0.02 (.09)_c_	0.34 (.21)_acd_	0.38 (.27)_bd_	576.65***
Duration of paternal migration	0.11 (.23)	0.01 (.07)_ab_	0.38 (.27)_ac_	0.02 (.08)_cd_	0.42 (.28)_bd_	558.88
Resident grandparent	24.3	16.11_ab_	22.73	30.29_ac_	81.69_bc_	161.58***
One or more siblings in household	81.38	85.85_ab_	90_cd_	69.71_ac_	59.15_bd_	83.15***
Mother completed upper secondary or higher education	35.78	34.17	51.21_a_	24.28_a_	42.25	58.33***
Household wealth						103.79***
Poorest	40.19	51.96_abc_	26.97_a_	32.64_b_	23.94_c_	
Medium	40.12	36.69	44.24	42.3	43.66	
Richest	19.69	11.34_abc_	28.79_a_	25.07_b_	32.39_c_	
Family process measures						
Family functioning	53.94	58.26_a_	55.76	44.65_a_	52.11	19.22***
Caregiver mental health problem	19.89	18.21	21.21	21.93	19.72	
*N*	1,498	714	330	383	71	

*Note*. Chi-squared tests were used for all comparisons except duration of maternal–paternal migration, which uses analysis of variance (ANOVA). Group differences are indicated by the same subscript based on Scheffe tests (ANOVA) and critical values of adjusted residuals for chi-squared. SR = self-report; CR = caregiver report.

*p < .05. **p < .01. ***p < .001.

### Concepts and Measures

Multivariate models included three sets of independent variables measuring characteristics of the household, characteristics of the child, and selected family processes. Five measures of child well-being based on child and caregiver reports were also included.

#### Household level

The household survey contained three household rosters to capture the complexity of transnational household structures: one for members currently residing in the household, a second for members of the household who were currently migrant, and a third “day roster” to record details about individuals who come into the household on a regular basis to provide care for children and/or adults within the primary household. Each of the rosters used the index child as the focal point for referencing relationships. This information was used to classify households into four groups according to *household migration status*: nonmigrant, father migrant, mother migrant, and both parents migrant. Children in nonmigrant households were taken as the reference group. A second set of migration measures, *duration of maternal and/or paternal migration*, indicates the proportion of a child’s lifetime the mother and/or father has worked abroad. Prior histories of parental migration to international destinations during the child’s lifetime are accounted for in the duration measures, including any history of prior migration by parents who are currently residing at home (the overall percentage of parents currently nonmigrant but with a prior history of international migration is 21%, with currently nonmigrant fathers 2 times more likely to have a prior history of migration than currently nonmigrant mothers). These variables were used to investigate whether there are any main or indirect effects of household migration status across different child well-being outcomes.

Two additional measures of household structure were also derived. The first was a binary indicator of whether a *grandparent resides in the household*. The second measure was an indicator of whether the *index child has one or more siblings* residing in the household. A further two measures summarized household socioeconomic status. The relative wealth of a household was measured by a *wealth index*. The index averages scores for housing quality, consumer durables and basic amenities, as devised for the Young Lives Project (http://www.younglives.org.uk) and recently used in a study of maternal mental health in four low income countries (De Silva, Huttly, Harpam, & Kenward, 2007). Following Filmer and Pritchett (2006), households were first grouped into five quintiles of relative wealth and then combined into tertiles where the first and second denote the poorest, the third and fourth denote medium wealth, and the fifth denotes the richest households. The quintiles were determined across the analytical sample, thus controlling for between-country variation in levels of wealth. Finally, a binary indicator of *completed maternal education* was constructed comparing those who had completed upper secondary or higher education with those with only primary or lower secondary education. Household wealth and maternal education were used to examine the impact of relative social status articulated in the second research question.

#### Child level

Two standard variables relating to characteristics of the index children were derived. Age was measured in *completed years*, and *sex of the child* was included where 1 denotes female. A measure of children’s *general knowledge about transnational households* was included based on the question, “Have you heard about (other) people who live around here going away to work in another country?” asked of all children responding to the older child questionnaire. The original question allowed for yes or no answers only. This measure was also used to examine the impact of relative social status on child well-being.

#### Family process

Two family process measures were constructed, one measure of *family functioning (APGAR)* and the other for *caregiver mental health*. The former is based on reports from the index children, and the latter on self-reports from the caregivers. APGAR is a rapid screening tool that has been widely used, including with Asian populations, to assess family functioning (Preechawong et al., 2007) and is scored on five questions about adaptation, partnership, growth, affection, and resolve (Austin & Huberty, 1989). The research scoring version was used, with five response categories (*never, hardly ever, some of the time, almost always, always*) and the components scores were summed to create a composite score for family functioning (range = 0 to 20); higher scores indicate better family functioning. The included measure was a binary indicator where 1 indicates the presence of positive family functioning (composite scores of 13 and above; Wolraich, Drotar, Dworkin, & Perrin, 2008).

A second family process measure was constructed for *caregiver mental health*. This was derived from the 20-item Self-Reporting Questionnaire (SRQ 20) which is recommended by the World Health Organization and widely used to screen for mental health problems in the developing world. It has been validated for Vietnam, along with many other countries and has been previously used in the region (Tuan, Harpham, & Huong, 2004). The suggested cutoff point of 7/8, with scores of 8 or more defining “cases,” was used to create a binary indicator of probable mental health problems (equal to 1) for caregivers. Following other studies, we expected that poor caregiver mental health was likely to impact negatively on child well-being (Goodman & Tully, 2006).

#### Well-being outcomes

The five measures were standardized as binary indicators to simplify the comparison between different indicators of well-being. Three of the five well-being outcomes were based on child reports, referred to as self-reported well-being measures. The first was a measure of *general happiness*. At the beginning of the survey the children were asked, “In general, are you happy or unhappy?” The question was carefully placed before questions on potentially sensitive topics, including parental absence, to avoid biasing the responses. Answers were elicited on a 5-point Likert scale (*very happy, happy, neither happy nor unhappy, unhappy, very unhappy*). For analysis, a binary variable was created where 1 indicates *very happy or happy*, and all other responses were coded as 0. The second outcome measure, *school enjoyment*, was created from responses to the question “In general, do you enjoy school?” Responses were also originally measured on a 5-point Likert scale (*never, hardly ever, some of the time, almost always, always*), and a binary indicator was constructed where 1 indicates *always or almost always*. This measure was included because school is an important social and public setting for children in middle childhood, and is the primary location where interaction with peers occurs. The third measure of self-reported child well-being was a subjective measure of a child’s own *school performance* compared to their peers, based on the question “How do your grades–marks compare to the grades–marks of your classmates?” and again measured on a 5-point Likert scale (*much better, better, about the same, worse, much worse*), with the created binary indicator equal to 1 for responses *much better* and *better*. This measure was selected as an additional indicator of the child’s sense of self-efficacy in the primary social environment.

Two further measures of child well-being based on reports from the child’s main caregiver were also included. Research Question 3 considers the extent to which children’s self-reported well-being differs from adult (caregiver) assessments. The CHAMPSEA older child and carer questionnaires do not contain identical questions on child well-being but we were able to construct measures from caregiver reports that closely match the child-reported measures. The first measure, *child is happy*, was based on caregiver ratings of the statement [the child is] “Often unhappy, downhearted or tearful” in relation to the index child in their care. Responses on a 3-point scale (*not true, somewhat true, certainly true*) were reverse coded to create a variable that reflected the level of happiness (i.e., absence of unhappiness). This question is part of the emotional subscale of the SDQ. Its use as a stand-alone measure is unconventional, but after careful consideration, we concluded that responses to this question were a close conceptual match to the child’s self-assessment of general happiness. A binary indicator was created with the *not true* response equal to 1. The second measure of caregiver-reported child well-being is a measure of *school performance*, which we take as comparable to the child’s self-assessed school performance relative to their peers. Answers to the question “What is the [index child’s] position in his–her class?” were elicited on a 3-point Likert scale (*above average, average, below average*) with 1 indicating *above average*.

The final column of Table 1 reports the significance of between-group differences across the four household types on each variable as determined by χ^2^ and analysis of variance tests. Post hoc tests were implemented when warranted. The distribution across household migration status groups is significant on most variables, with child age and sex as notable exceptions most likely explained by the sampling strategy, which targeted a narrow age range and ensured approximately equal numbers of girls and boys.

#### Analysis

The analyses used hierarchical logistic regression modeling to examine the effects of parental migration status, household characteristics and family processes across different measures of child well-being. All analyses were completed using StataIC 11 and the results are reported as odds ratios with confidence intervals. Log likelihood values are included in the tables as indicators for model specification following Greene (2000) and Borooah (2002). Each of the measures of child well-being was regressed on parental migration status, a set of control variables (index child age and sex) and two sets of covariates (socioeconomic status and household structure, and family process measures). The five outcome measures were all dichotomized into binary form to allow for simpler comparison between child- and caregiver-reported measures and also based on the distribution of the measures across and within countries.

Two other considerations should be noted about the analytical strategy. First, the pairwise correlations for the five outcome measures were calculated and as they were all 0.10 or lower, with the exception of the correlation between self- and caregiver report of school performance at 0.25, there was no reason for concern that the analytical results were purely due to chance. Second, because of the sampling design, parents in one fifth of the households currently nonmigrant had a history of prior migration during the child’s lifetime. All of the final models were run after dropping these cases to examine the robustness of the migration parent classifications. Sensitivity tests were conducted by rerunning all of the analyses after dropping the 153 cases where either the mother (50 cases) or father (103 cases) had been a previous international migrant but was not a migrant at the time of survey to ascertain the robustness of the group comparisons (models not shown). As the results were very similar for both sets of analyses, we concluded that the use of current household migration status groups was valid for comparison.

## Results

In general, children reported positive well-being for both general happiness and school enjoyment. Nevertheless, the bivariate differences (see Table 1) were statistically significant, with children in migrant mother households less likely to report overall general happiness compared to those living in nonmigrant households. In contrast, the measure of school enjoyment was uniformly high across all parental migration statuses with no significant differences. The proportion of happy children based on caregiver reports is considerably lower overall compared to the proportion derived from child self-reports, although children in migrant mother households were still assessed as less happy compared to those in nonmigrant households. Neither child nor caregiver assessments of school performance showed statistically significant differences among household types. In general, caregivers report higher school performance compared to the children themselves.

The test of group differences for knowledge of other transnational households was also statistically significant, with children in migrant father households more likely to report knowledge of other transnational households compared to children in nonmigrant households. This is supported by the previous literature that suggests that mothers who stay in sending countries may facilitate interaction with other transnational households for their children (Parreñas, 2005). The next set of variables included resident grandparent, presence of sibling(s), maternal education, and household wealth. The overall number of households with a resident grandparent was a bit less than one third, which is interesting given the common perception of larger extended families in Asia (Frankenberg et al., 2002; Ofstedal, Knodel, & Chayovan, 1999), although some recent research indicates changing family forms in the region (Chu, Xie, & Yu, 2011). Both parents migrant households, not surprisingly, were much more likely to have a resident grandparent compared to other household types. Migrant mother households were also more likely to have a resident grandparent compared to nonmigrant households, providing some indication of changes in caregiving arrangements after the migration of a child’s “usual caregiver,” the mother (the proportion of children being cared for by mothers in nonmigrant households is 87% across the analytical sample). Compared to migrant father households, both parents migrant households were significantly less likely to have more than one coresident dependent child. The wealth profile of the households shows several differences across household types, with a higher proportion of all transnational household types in the richest category. Among the family process measures, there were only statistically significant differences for child report of family functioning, with children in migrant mother households less likely to report positive family functioning compared to children in nonmigrant households.

The bivariate results highlighted some interesting differences between children living in different types of household. However, multivariate models were required to test the combined effects of the independent variables on child well-being outcomes and to examine the research questions. Multilevel models were not fitted because, with only three countries in the sample, the number of units at the second level is too small for adequate specification. Implementing a Huber-White correction (as multilevel models do) will generally increase the size of the standard errors; thus, standard errors may be biased downward, which can lead to rejection of the null hypothesis erroneously (Greene, 2000) and this should be considered in assessment of the multivariate models. Country dummy indicators were included as fixed effects to account for unobserved differences at the country level. Discussion of significant differences among the three study countries is limited as these differences are not the focus of the current inquiry. Extracts from the final models including all control and covariates are presented in Table 2 and the full hierarchical model for selected outcomes is available in Table 3.

**Table 2:** Child Well-Being Measures—Fully Adjusted Models

	General happiness						School performance						School enjoyment		
			
	Self-report			Caregiver report			Self-report			Caregiver report			Self-report		
					
	95% CI			95% CI			95% CI			95% CI			95% CI		
					
Measures	OR	LL	UL	OR	LL	UL	OR	LL	UL	OR	LL	UL	OR	LL	UL
Nonmigrant															
Father migrant	0.54	0.34	0.85**	0.66	0.44	1.00*	1.44	0.97	2.13	1.16	0.78	1.72	1.27	0.77	2.25
Mother migrant	0.33	0.21	0.51***	0.49	0.33	0.72***	1.26	0.86	1.84	1.15	0.79	1.68	0.91	0.49	1.34
Both migrant	0.34	0.15	0.75**	0.61	0.29	1.27	1.20	0.60	2.42	1.52	0.76	3.03	0.72	0.33	2.15
Duration of:															
Maternal migration	5.06	1.81	14.15**	3.39	1.36	8.45**	0.68	0.30	1.56	0.39	0.17	0.90*	1.53	0.51	4.75
Paternal migration	1.23	0.52	2.91	1.74	0.80	3.80	0.73	0.35	1.53	1.14	0.55	2.37	0.49	0.18	1.13
Low wealth															
Medium wealth	1.30	0.95	1.78	0.95	0.72	1.25	1.05	0.80	1.37	1.23	0.94	1.61	0.93	0.64	1.31
High wealth	1.30	0.85	1.98	1.01	0.70	1.46	1.14	0.80	1.63	1.46	1.02	2.08*	0.84	0.53	1.39
Mother’s education secondary or more	0.94	0.65	1.34	1.49	1.10	2.03**	1.50	1.10	2.03**	1.55	1.13	2.11**	1.66	1.08	2.44*
Knowledge of transnational households	0.81	0.57	1.13	1.18	0.89	1.56	1.22	0.91	1.63	1.24	0.93	1.64	1.67	1.16	2.30**
Family functioning	1.26	0.97	1.64	1.19	0.94	1.50	1.28	1.02	1.61*	1.45	1.15	1.81**	1.77	1.36	2.49***
Caregiver mental health	0.80	0.58	1.11	0.33	0.25	0.44***	0.74	0.55	1.00*	1.02	0.77	1.36	0.86	0.56	1.17

*Note*. All models include indicators of index child age and sex, household structure, and country of residence. OR = odds ratio; CI = confidence interval.

* *p* < .05.

** *p* < .01.

*** *p* < .001.

**Table 3:** General Happiness and Well-Being

	Self-report									Caregiver-report								
		
	Model A			Model B			Model C			Model D			Model E			Model F		
						
	95% CI			95% CI			95% CI			95% CI			95% CI			95% CI		
						
	OR	LL	UL	OR	LL	UL	OR	LL	UL	OR	LL	UL	OR	LL	UL	OR	LL	UL
Indonesia																		
The Philippines	1.05	0.75	1.48	0.95	0.63	1.43	0.92	0.61	1.39	0.84	0.63	1.12	0.67	0.48	0.94*	0.60	0.42	0.85*
Vietnam	0.81	0.59	1.12	0.77	0.55	1.07	0.73	0.53	1.02	0.20	0.15	0.26***	0.21	0.16	0.27***	0.17	0.12	0.23***
Child age																		
9																		
10	0.90	0.65	1.23	0.91	0.66	1.25	1.08	0.66	1.24	1.06	0.82	1.38	1.09	0.84	1.42	1.08	0.82	1.41
11	0.77	0.56	1.07	0.76	0.55	1.06	0.77	0.55	1.07	1.22	0.92	1.61	1.24	0.93	1.65	1.30	0.97	1.74
Child is girl	1.10	0.84	1.42	1.10	0.85	1.43	1.08	0.83	1.41	0.84	0.67	1.05	0.85	0.68	1.06	0.81	0.64	1.01
Nonmigrant																		
Father migrant	0.53	0.34	0.83**	0.53	0.34	0.82**	0.54	0.34	0.85**	0.63	0.43	0.93*	0.61	0.41	0.90*	0.66	0.44	1.00*
Mother migrant	0.32	0.21	0.49***	0.31	0.20	0.48***	0.33	0.21	0.51***	0.45	0.30	0.66***	0.44	0.29	0.64**	0.49	0.33	0.72***
Both migrant	0.32	0.15	0.67**	0.33	0.15	0.73**	0.34	0.15	0.75**	0.60	0.30	1.19	0.53	0.26	1.10	0.61	0.29	1.27
Duration of:																		
Maternal migration	5.49	2.01	14.98**	5.08	1.81	14.22**	5.06	1.81	14.16**	4.32	1.82	10.29**	3.51	1.43	8.61**	3.39	1.36	8.45**
Paternal migration	1.39	0.60	3.21	1.24	0.53	2.91	1.23	0.52	2.91	2.04	0.97	4.30	1.79	0.83	3.85	1.74	0.80	3.80
Low wealth																		
Medium wealth				1.34	0.98	1.84	1.30	0.95	1.78				1.09	0.83	1.42	0.95	0.72	1.25
High wealth				1.35	0.89	2.05	1.30	0.85	1.98				1.18	0.83	1.70	1.01	0.70	1.46
Mother’s education secondary or more				0.95	0.66	1.36	0.94	0.65	1.34				1.54	1.14	2.08**	1.49	1.10	2.03**
Knowledge of transnational households				0.82	0.59	1.15	0.81	0.57	1.13				1.27	0.97	1.67	1.18	0.89	1.56
Resident grandparent				1.10	0.79	1.52	1.09	0.79	1.51				1.06	0.80	1.40	1.03	0.77	1.37
One or more siblings in household				1.39	0.97	1.97	1.40	0.98	2.00				0.78	0.57	1.08	0.81	0.59	1.13
Family functioning							1.26	0.97	1.64							1.19	0.94	1.50
Caregiver mental health							0.80	0.58	1.11							0.33	0.25	0.44***
−log likelihood	−711.205			−707.363			−705.030			−909.043			−899.618			−869.762		

*Note*. OR = odds ration; CI = confidence interval.

* *p* < .05.

** *p* < .01.

*** *p* < .001.

### Question 1: Is Maternal and/or Paternal Migration Associated With Decreased Child Well-Being?

The first research question addresses the popular concern about decreased child well-being among children residing in migrant mother households. The empirical analysis centred on the examination of two dimensions of parental migration, gender of migrant parent(s), and the proportion of the index child’s lifetime that the mother and the father had been an international migrant. The pattern of responses was not uniform across the different dimensions of child well-being. When the general happiness of children was considered (Table 2), children in all transnational households were disadvantaged compared to their peers living in nonmigrant households. Children of migrant mothers were the least likely to be assessed as generally happy, based on both self- and caregiver reports. The duration of maternal migration was also an important factor associated with the general well-being of children, though not always in the expected direction. Children who have spent a larger proportion of their lifetime living in migrant mother households were much more likely to be recorded as generally happy. Notably, there was also evidence of a paternal migration effect *as well as* a maternal migration effect, and this was particularly true when children self-report on general happiness. After accounting for all the covariates, children were less likely to see themselves as happy across all transnational household types. The relative effect size of the indicator “migrant mother household” was larger than for the comparable indicator “migrant father household,” and the duration of time fathers have spent abroad was not a significant predictor based on either caregiver or self-report. There was, additionally, no evidence of any main migration effects for the other dimensions of child well-being considered here (Table 2). School performance based on caregiver report did indicate a secondary migration effect for duration of maternal migration, with longer duration of maternal migration exerting a negative influence on index child school performance. There was, thus, evidence that children of migrant parents were disadvantaged in well-being, although this association was sensitive to the dimension of well-being that is measured. Children across all transnational household types were less happy compared to those in nonmigrant households, and there was some indication that children in migrant mother households are relatively more disadvantaged than children in other types of transnational household.

### Question 2: Does the Relative Social Status of Children Moderate the Influence of Transnational Household Status on Child Well-Being?

The second research question draws on theoretical constructs about well-being of immigrant children in relation to peers (García Coll & Szalacha, 2004) to suggest that changes in social status, in particular, higher levels of socioeconomic status (primarily indicated by relative wealth) associated with parental migration, could promote resilience among left-behind children. Additionally, recognizing that isolation from similar “others” could act as a drain on positive child well-being, we examined another proxy measure of social status, whether the child has knowledge of (other) transnational households. A series of interaction models (not shown) was fitted to examine the moderating relation by interacting the measures of social status with the migration measures. None of the interaction terms between social status and migration status (household migration status, duration away) was significant, and model fit tests did not indicate an improvement in model fit for any of the interacted models. Moreover, the stepwise hierarchical models did not show changes in coefficients related to parental migration status with the addition of the social status measures. We therefore conclude that there was no direct evidence of a moderating relation between social and migration status.

There was, however, demonstration of direct effects between social status and child well-being. Across the different dimensions of child well-being, one measure of socioeconomic status, maternal completed education, was the most consistently significant predictor and is associated with a greater likelihood of child happiness, enjoyment of school, and good school performance. Despite a bivariate relation between household wealth and household migration status, with higher wealth observed for all transnational household types, there was little evidence that differences in household wealth contribute to differences in general happiness and school enjoyment. There was some confirmation that household wealth contributes to academic performance (in the eyes of the caregiver), though this is only for children in the wealthiest households (Table 2).

The second aspect of social status, knowledge of (other) transnational households’ knowledge of similar others, was significantly related to one dimension of child well-being, self-reported school enjoyment. Children’s knowledge of (other) transnational families within the community was associated with higher levels of school enjoyment. All children, regardless of parental migration status, were more likely to report enjoyment in the wider community of school if they know people who live in transnational families.

### Question 3: To What Extent Do Caregiver and Child Assessments of Child Well-Being Differ?

The third research question addresses the possibility that adult perceptions of child well-being (from the caregivers of the index children in this instance) may differ from child perceptions of their own well-being. It may be, for example, that adults are more influenced by cultural and social norms that prescribe gendered role divisions for reproductive and productive labor within families (Parreñas, 2001, 2010). Comparison of two pairs of similar self- and caregiver-reported measures (general happiness and school performance) is used to examine this question. In general, the pattern of association between household migration status and each set of child well-being measures was very similar (Table 2). As noted in the discussion for Research Question 1, a main migrant parent effect was only observed for general happiness, with children in mother migrant households least likely to be assessed as happy based on either self- or caregiver report. However, there was also evidence of a *paternal* migration effect negatively associated with child well-being for self- and caregiver reports. The effect size for all transnational household types was larger for child self-report, suggesting that the impact of parental absence is, perhaps, felt more acutely from the child’s perspective. There was therefore no evidence to suggest significant divergence between caregiver and self-assessment of child happiness beyond the difference in the magnitude of the effect.

The second set of paired measures (Table 2) showed some evidence of a divergence between self- and caregiver report of child well-being as measured by school performance. Children who have experienced a longer duration of maternal migration during their lifetime were less likely to be assessed as performing better than their peers at school by their caregivers, but this is not replicated for child self-report of school performance. This finding provides an indication that caregivers may view children who experience a longer duration of maternal absence overseas as particularly disadvantaged in school performance.

Beyond the migration effects, the contribution of the family process measures to explaining variation in child well-being was important. Child assessment of family functioning is an important positive explanatory measure across well-being measures, contributing to increased school enjoyment and school performance. Interim models (not shown) indicated that family functioning was also an important predictor of child self-reported happiness before controlling for the duration of parental migration. In addition, caregiver mental health was an important predictor of child well-being, most noticeably associated with significantly decreased happiness based on caregiver report (but not with caregiver assessment of school performance). This was not reflected in the children’s self-reported general happiness and may partly be due to caregivers with a probable mental health condition being less likely to have a positive outlook in general. More worrisome was the association between caregiver mental health and children’s self-reported school performance, as children in the care of those with poorer mental health were less likely to see *themselves* as high performers at school, suggesting that children may be internalizing effects of poor caregiver mental health.

In response to our third research question on the extent to which child and caregiver reports of child well-being differ, it was apparent that the answer depends on which dimension of well-being was investigated. There was little evidence to suggest a divergence in assessments by children and caregivers of general happiness, but the analysis did suggest a divergence in assessments of school performance relative to the duration of maternal absence. This finding merits further research.

In sum, the study findings provide insights into all three research questions. In addressing Research Question 1, we found evidence for decreased well-being of children of migrants—both those of migrant mothers *and* migrant fathers—for general happiness, and, based on the duration of maternal absence, for caregiver-assessed school performance. In answer to Research Question 2, our results underscore the importance of social status but not as moderating the association between parental migration and child well-being. Across the measured dimensions of well-being, indicators of social status (in particular maternal education) and family process (including caregiver mental health and family functioning), are consistently associated with variations regardless of parental migration status. Research Question 3 asks about divergence between child and caregiver assessment across the paired measures of well-being, and the findings highlight the importance of considering multiple dimensions when investigating the relation between parental migration and child well-being, as results are similar for measures of general happiness, but diverge when considering child and caregiver assessments of school performance.

## Discussion

Participation in international labor migration is an increasingly common solution to household livelihood attainment for millions of families across the world. As more women, and mothers, have joined these global circuits of labor migration, public and academic attention has illuminated concerns about child well-being for families who are seeking to better the life chances of the next generation. Although these children do experience separation and loss, there is some reason to believe that remaining in countries of origin may offer several advantages as the children usually remain embedded within familiar family and community networks and are bolstered by overseas remittances. Nevertheless, children left behind are also a potentially vulnerable population, lacking intimate contact with their migrant parent(s). The empirical evidence to date is mixed. This article has examined different dimensions of well-being for children of international migrant parents in one of the major labor-sending region of the world, South-East Asia. We focused on the examination of three related research questions, and considered some existing theory on immigrant child development to guide our conceptualization and analysis.

Within the transnational context, we questioned whether maternal and/or paternal overseas migration is associated with decreased well-being for children left behind. Other studies had found decreased well-being among children in migrant mother households. Children in migrant mother households *were* less happy compared to those living with nonmigrant parents, but children across *all* transnational household types were also less happy, especially in the eyes of the children themselves. The lack of greater explanatory power in the full set of predictors for general happiness suggests a more internal process that is not attenuated by other measures of household structure and family process, echoing an emotional vulnerability to parental absence found in previous research from Latin America and the region (ECMI–CBCP/AOS–Manila et al., 2004). The negative relation between caregiver mental health and child well-being are troubling (caregiver report of child happiness and self-report of school performance). Previous research has indicated a tendency for caregiver reports to perceive child psychological well-being through the lens of parental (or caregiver) depression (Goodman & Tully, 2006; Suarez-Orozco & Suarez-Orozco, 2001) and more nuanced study is needed to understand better how caregiver mental health influences child development in transnational households. On a more promising note, there is some evidence that, over time, children in migrant mother households develop resilience, adapting to change in family configuration, as indicated by the positive relation between duration of maternal migration and happiness. This is in contrast to findings from research in China (Fan et al., 2010), although the cited study did not examine the gender of the migrant parent and thus is not directly comparable. However, other research indicates that internal migrants in general tend to be less well off than international migrants (Adams & Page, 2005) and this may be a bias inherent to comparisons between this study and Chinese research on migration. Future research should examine the relation between duration of time away and child well-being to provide guidance for community support and intervention to children in migrant mother households during critical developmental years of early and middle childhood.

While immigrant children exhibit decreased advantage over time, we found that children who spend a longer proportion of their lifetime residing in migrant mother households were happier, suggesting that time is protective for these children. Further research into adolescence, young adulthood, and family formation of the next generation is needed to understand the longer term effects of parental overseas migration, and such longitudinal research would provide greater specification about whether the loss of maternal caregiving is, on balance, worth the gains in material advantage.

Among studies of immigrant children residing in host countries one frequently observed phenomenon is resilience as measured by psychological well-being despite significantly lower income, which is the reverse of the association for native-born ethnic and economic minorities (García Coll & Szalacha, 2004). Drawing on this, we questioned whether the increased wealth of children living in transnational households compared to their peers in country of origin could be a protective factor for well-being. We found no evidence that social status operates through the migration status of parents to predict child well-being. There was little evidence of a wealth effect (either negative of positive) on well-being with the exception of caregiver-reported school performance. Wealth does not seem to be an important factor contributing to child well-being on these particular constructs despite significant bivariate differences with household migration status. This finding indicates that *material gains* from migration do not impact on the dimensions of child well-being measured in this study, and therefore provides no support for Parreñas’s (2001) claim that the material security afforded by migration may be an influential factor contributing to the luxury of demanding greater emotional security.

We also considered that the social status of the child could be influenced through identification as a child of a migrant parent. This conceptualization of social status is an extension of the theoretical model explaining the influence of negative socialization processes on child well-being (García Coll & Szalacha, 2004). In this study, greater knowledge of other transnational households was expected to act as a protective factor buffering negative socialization processes associated with parental migration. This measure was found to be an important positive influence on only one dimension of child well-being, self-reported enjoyment of school, but there was no evidence that children enjoy school differentially based on whether or not the household has a migrant parent. All children, whether they are members of transnational households or not, were more likely to say they enjoy school if they know people who are members of a transnational household. The absence of a “migrant parent” advantage in reported school performance is perhaps surprising given that many migrant parents emphasize the education of their children when explaining the rational for their overseas migration. Nevertheless, the possibility that the presence of transnational households in a community may exert a positive effect on school engagement and impact indirectly on school performance clearly warrants future research. One of the macroeconomic rationales for labor migration as a form of development is the idea of positive spillover to origin communities (Adams & Page, 2005). This finding provides some evidence of such a spillover, although given the cross-sectional nature of the survey data, causal ordering cannot be determined with certainty.

We further sought to identify differences between self-report and caregiver report of child well-being by comparing child and caregiver responses on two sets of similar questions. Given public concerns about maternal absence within sending countries such as the Philippines, we expected that adult caregivers might be more susceptible to the influence of cultural norms idealizing maternal care in their assessments of the well-being of children in their care. This did not appear to be the case. Indeed, the relative negative effect of maternal migration on child happiness was greater based on the child’s self-report, suggesting that caregivers may, in fact, under-report child distress due to parental migration. Children may be in greater distress than assessed by their caregiver, which is worrisome. Additionally, the children may already have internalized some of the negative self-conceptualizations from the wider social community (Parreñas, 2010) and future research should consider how best to incorporate dimensions of community characteristics to capture cultural and social norms that may influence child well-being.

The inclusion of the child’s perspective is an important contribution of this study illustrating, in particular, how children differentiate across dimensions of well-being. More negative assessments of general happiness were associated with parental absence, highlighting a perception of vulnerability and emotional loss. The APGAR measure of family functioning was positively associated with both school enjoyment and self-appraisal of performance, and this emphasizes the importance of healthy family functioning in promoting child well-being regardless of parental migration.

Although not the focus of this analysis, differences based on CHAMPSEA study country also suggest the importance of distal influences (see Table 3). While the negative backlash against maternal migration in the Philippines has been documented, the magnitude of the effect for the Vietnamese sample suggests that caregivers’ perspective on maternal migration is an important determinant of variation in caregiver-reported child happiness, with these Vietnamese children much less happy compared to children in the comparison country, Indonesia. Rotation of reference groups (models not shown) illustrates that these differences are robust when the Philippines sample is used as the comparison group. Even though the influence of socialism reduced some elements of gender inequality in Vietnam, the influences of Confucianism and patriarchal lineage underpin persistent gender inequalities that may be reflected in the findings of this study.

### Concluding Remarks

This study is not without limitations. As with all cross-sectional studies, casual pathways cannot be delineated, and as such the findings should be considered exploratory. Delineating causal pathways awaits longitudinal data collection. As the country samples are not nationally representative, the findings cannot be generalized to all children within a particular country or to the region as a whole. The sample was drawn from areas of high international out-migration in each study country and therefore may not accurately reflect other migrant populations. The observed differences, for socioeconomic status for example, might be more marked if compared to children in areas of lower out-migration.

This study has, however, made important contributions to understanding child well-being among children in transnational households left-behind in sending countries despite these limitations. What is especially noteworthy is that while both children and caregivers report particular disadvantage for children in migrant mother households, there is also evidence of a *more universal migrant disadvantage*. In our previous study using the same source data from the CHAMPSEA project and standardized measures of child psychological well-being, we found evidence of contextually-based migrant parent effects, including a disadvantage for children of migrant fathers in some study countries varying according to which dimension of psychological well-being was measured (Graham and Jordan, 2011). The current study has confirmed the sensitivity of the relation between parental migration and child well-being to the dimension of child well-being measured and, at least in the case of subjective measures, whether the child or caregiver is reporting on the child.

Child well-being as measured by general happiness is most sensitive to parental migration, and the responses of children and caregivers in the CHAMPSEA study are quite similar. Children in transnational households were less happy compared to their peers in nonmigrant households, possibly reflecting the experience of pain and loss due to parental absence and in particular maternal absence. This supports extant research on how children in other regions such as Latin America experience parental absence, although our findings also suggest a qualification because longer durations of maternal absence appear to be associated with resilience in left-behind children. Children do not experience any deficit in positive self-assessment of school performance nor in enjoyment of school activities based on parental migration. In fact, there appears to be a positive influence of transnational household configurations, with children in the study communities reporting higher levels of school enjoyment whether they reside in a nonmigrant or transnational household. Finally, this study has illustrated how children are more sensitive to proximate influences such as family functioning and caregiver mental health in their day-to-day functioning than they are, perhaps, to parental absence.
